# Development of a Rapid and Fully Automated Multiplex Real-Time PCR Assay for Identification and Differentiation of *Vibrio cholerae* and *Vibrio parahaemolyticus* on the BD MAX Platform

**DOI:** 10.3389/fcimb.2021.639473

**Published:** 2021-02-25

**Authors:** Zhenpeng Li, Hongxia Guan, Wei Wang, He Gao, Weihong Feng, Jie Li, Baowei Diao, Hongqun Zhao, Biao Kan, Jingyun Zhang

**Affiliations:** ^1^ State Key Laboratory of Infectious Disease Prevention and Control, National Institute for Communicable Disease Control and Prevention, Chinese Center for Disease Control and Prevention, Beijing, China; ^2^ Wuxi Center for Disease Control and Prevention, Wuxi, China; ^3^ Collaborative Innovation Center for Diagnosis and Treatment of Infectious Diseases, Hangzhou, China

**Keywords:** *Vibrio cholerae*, *Vibrio parahaemolyticus*, multiplex, real-time PCR, BD MAX

## Abstract

*Vibrio cholerae* and *Vibrio parahaemolyticus* are common diarrheal pathogens of great public health concern. A multiplex TaqMan-based real-time PCR assay was developed on the BD MAX platform; this assay can simultaneously detect and differentiate *V. cholerae* and *V. parahaemolyticus* directly from human fecal specimens. The assay includes two reactions. One reaction, BDM-VC, targets the gene *ompW*, the cholera toxin (CT) coding gene *ctxA*, the O1 serogroup specific gene *rfbN*, and the O139 serogroup specific gene *wbfR* of *V. cholerae*. The other, BDM-VP, targets the gene *toxR* and the toxin coding genes *tdh* and *trh* of *V. parahaemolyticus*. In addition, each reaction contains a sample process control. When evaluated with spiked stool samples, the detection limit of the BD MAX assay was 195–780 CFU/ml for *V. cholerae* and 46–184 CFU/ml for *V. parahaemolyticus*, and the amplification efficiency of all genes was between 95 and 115%. The assay showed 100% analytical specificity, using 63 isolates. The BD MAX assay was evaluated for its performance compared with conventional real-time PCR after automated DNA extraction steps, using 164 retrospective stool samples. The overall percent agreement between the BD MAX assay and real-time PCR was ≥ 98.8%; the positive percent agreement was 85.7% for *ompW*, 100% for *toxR*/*tdh*, and lower (66.7%) for *trh* because of a false negative. This is the first report to evaluate the usage of the BD MAX open system in detection and differentiation of *V. cholerae* and *V. parahaemolyticus* directly from human samples.

## Introduction

*Vibrio cholerae* is the etiological pathogen of cholera, an acute watery diarrheal disease. Researchers have estimated that in endemic countries globally, there are approximately 1.3 billion people at risk for cholera, 1.3–4.0 million cholera cases occurring annually, and 21,000–143,000 deaths among these cases (Ali et al., 2015). In 2017, more than 1.2 million cases and 5,654 deaths were reported from 34 countries (WHO, 2018). The current seventh cholera pandemic continues to be a major public health threat for countries in Asia, Africa, and the Americas (WHO, 2018). With more than 220 serogroups of *V. cholerae*, only the O1 and O139 serogroups have been associated with epidemics and pandemics (Reidl and Klose, 2002; Mahapatra et al., 2014; Bonnin-Jusserand et al., 2019). Cholera toxin (CT) encoded by *ctxA* and *ctxB* is responsible for severe, cholera-like diseases in epidemic and sporadic forms (Holmgren, 1981; Wernick et al., 2010). In assessing the public health significance of an isolate of *V. cholerae*, the possession of the O1 or O139 antigen is a marker of epidemic or pandemic potential (Kaper et al., 1995), and the production of CT is one of the most important properties to be determined.

*V. parahaemolyticus* causes acute gastroenteritis mostly associated with the consumption of raw or improperly cooked contaminated seafood (Daniels et al., 2000). It often leads to sporadic cases or outbreaks in coastal areas during warm seasons (Baker-Austin et al., 2017) and has been a major seafood-borne pathogen and a global public health concern. Thermostable direct hemolysin (TDH, encoded by the gene *tdh*) and TDH-related hemolysin (TRH, encoded by the gene *trh*) are considered to be the main pathogenic factors of *V. parahaemolyticus* (Shinoda, 2011). The genes *tdh* and *trh* exist in most clinical isolates and are relatively rare in environmental isolates (Theethakaew et al., 2013).

Timely detection of *V. cholerae* and *V. parahaemolyticus* infection in patients with diarrhea, as well as identification of serogroups and virulence factors, is of great significance for patient treatments and controlling disease spread. With the development of molecular detection technology, various PCR-based detection methods have been developed and applied, including conventional PCR, real-time PCR, and multiplex PCR (Tada et al., 1992; Rivera et al., 2003; Jeyasekaran et al., 2011; Tebbs et al., 2011; Tall et al., 2012). These methods require that before amplification, nucleic acids be extracted independently, which is time-consuming and labor-intensive. Therefore, there is an urgent need for an automated and integrated platform to complete the molecular detection of *Vibrios* directly from infected patients’ specimens.

The BD MAX system (Becton Dickinson Inc., Maryland, USA) is a fully automated molecular platform for *in vitro* diagnostic, as well as in-house-developed tests. The platform extracts DNA or RNA using specific extraction reagents, followed by real-time PCR amplification and detection of fluorescence in up to five channels. The system can be run in an open mode that allows adding any user-specific primers and PCR reagents. In this study, we developed a multiplex real-time PCR assay on a BD MAX open system for detection and differentiation of *V. cholerae* and *V. parahaemolyticus* directly from human fecal specimens.

## Materials and Methods

### Strains and Samples

*V. cholerae* strains N16961 (O1 serogroup, CT positive) (Heidelberg et al., 2000) and ATCC 51394 (O139 serogroup, CT positive), and *V. parahaemolyticus* strain VP8 (a clinical strain, *tdh* and *trh* positive) were used as positive reference strains for the establishment of the real-time PCR assay. The 63 strains (**Table 1**) used for specificity evaluation included 22 V*. cholerae* strains (11 O1 serogroup and 11 O139 serogroup), 19 V*. parahaemolyticus* strains, 8 diarrheagenic *Escherichia coli* (DEC), 6 *Salmonella* spp., 2 *Shigella* spp., 1 V*. mimicus*, 1 V*. fluvialis*, 1 V*. vulnificus*, 1 V*. anguillarum*, 1 *Plesinomonas shigelloides*, and 1 *Aeromonas hydrophila.* The *ctxA* gene of *V. cholerae* and the *tdh*/*trh* genes of *V. parahaemolyticus* were screened and determined by singleplex real-time PCR assays as described in the “analysis of clinical samples” section below with primers/probes listed in **Table 2**.

**Table 1:** Strains used in the study.

	Strain	Year	Description
***Vibrio cholerae* (n = 22)**	N16961	1975	O1 Inaba, reference strain, *ctxA*+
	ICDC-VC4679	1977	O1 Ogawa, clinical strain, *ctxA*+
	ICDC-VC4684	1978	O1 Ogawa, clinical strain, *ctxA*+
	ICDC-VC4685	1978	O1 Ogawa, clinical strain, *ctxA*+
	ICDC-VC4689	1978	O1 Ogawa, clinical strain, *ctxA*+
	ICDC-VC4692	1980	O1 Inaba, clinical strain, *ctxA*+
	ICDC-VC4696	1991	O1 Inaba, clinical strain, *ctxA*+
	ICDC-VC4879	1988	O1 Ogawa, *ctxA*+
	ICDC-VC4981	1982	O1 Inaba, clinical strain, *ctxA*+
	ICDC-VC4670	2008	O1 Inaba, estuarine water, *ctxA*-
	ICDC-VC4876	1987	O1 Ogawa, clinical strain, *ctxA*-
	ATCC 51394	1992	O139, reference strain, *ctxA*+
	ICDC-VC206	2001	O139, clinical strain, *ctxA*+
	ICDC-VC213	2003	O139, clinical strain, *ctxA*+
	ICDC-VC495	2005	O139, clinical strain, *ctxA*+
	ICDC-VC818	2003	O139, clinical strain, *ctxA*+
	ICDC-VC1193	1997	O139, clinical strain, *ctxA*+
	ICDC-VC1662	2006	O139, clinical strain, *ctxA*+
	ICDC-VC2384	2009	O139, clinical strain, *ctxA*+
	ICDC-VC2650	1994	O139, clinical strain, *ctxA*+
	ICDC-VC207	2002	O139, clinical strain, *ctxA*-
	ICDC-VC3768	1993	O139, clinical strain, *ctxA*-
***V. parahaemolyticus* (n = 19)**	VP8	1984	Clinical strain, *tdh*+, *trh*+
	VP6-1	1984	Clinical strain, *tdh*+, *trh*-
	VP649	2010	Clinical strain, *tdh*+, *trh*-
	VP669	2011	Clinical strain, *tdh*+, *trh*-
	VP651	2010	Clinical strain, *tdh*+, *trh*-
	VP652	2010	Clinical strain, *tdh*+, *trh*-
	VP667	2011	Clinical strain, *tdh*+, *trh*-
	VP668	2011	Clinical strain, *tdh*+, *trh*-
	VP670	2011	Clinical strain, *tdh*+, *trh*-
	VP674	2011	Clinical strain, *tdh*+, *trh*-
	VP686	2011	Clinical strain, *tdh*+, *trh*-
	VP654	2010	Aquatic product, *tdh*-, *trh*-
	VP656	2010	Aquatic product, *tdh*-, *trh*-
	VP660	2010	Aquatic product, *tdh*-, *trh*-
	VP661	2010	Aquatic product, *tdh*-, *trh*-
	VP678	2011	Aquatic product, *tdh*-, *trh*-
	VP680	2011	Aquatic product, *tdh*-, *trh*-
	ATCC17802	NA	Reference strain, *tdh*-, *trh*-
	Vp-8411	NA	Environmental strain, *tdh*-, *trh*-
**Non-target species**			
*V. mimicus* (n = 1)	SX-4	2009	Clinical strain, *ctxA*+
*V. fluvialis* (n = 1)	CICC21612	NA	Reference strain
*V. vulnificus* (n = 1)	ATCC27562	1979	Estuarine. Reference strain
*V. anguillarum* (n = 1)	ATCC17749	NA	Clinical strain
*Plesinomonas shigelloides* (n = 1)	PS6	2012	Clinical strain
*Aeromonas hydrophila* (n = 1)	AH1	2012	Clinical strain
diarrheagenic *Escherichia coli* (n = 8)	EPEC49	2013	Clinical strain
	EPEC51	2013	Clinical strain
	EPEC87	2013	Clinical strain
	EAEC68	2013	Clinical strain
	EAEC73	2013	Clinical strain
	ETEC42	2013	Clinical strain
	EIEC9	2013	Clinical strain
	CN-ETEC-16	2016	Clinical strain
*Salmonella* (n = 6)	St1787	2002	*S. typhi*, clinical strain
	CT18	1993	*S. typhi*, clinical strain. Reference strain
	St1806	2016	*S. typhi*, clinical strain
	St1866	2016	*S. typhi*, Sewage
	St1868	2016	*S. typhi*, clinical strain
	Sa10387	2013	*S. enteritidis*, clinical strain
*Shigella* (n = 2)	4153-6	2012	Clinical strain
	CN-Shigella-2	2016	Clinical strain

**Table 2:** Primers and probes for detection of virulence genes in strains and clinical samples.

Target genes	Code	Sequence (5’-3’)	size (bp)
*ctxA*	CT1 F	CTTCCCTCCAAGCTCTATGCTC	114
	CT1 R	TACATCGTAATAGGGGCTACAGAG	
	CT1 P	FAM-ACCTGCCAATCCATAACCATCTGCTGCTG-BHQ1	
*tdh*	TDH F	AATGGTTGACATCCTACATGACTG	100
	TDH R	TTTACGAACACAGCAGAATGACC	
	TDH P	FAM-TATAGCCAGACACCGCTGCCATTGTATAGT-BHQ1	
*trh*	TRH F	GATTGCGTTAACTGGTGATTCAG	105
	TRH R	GCGATTGATCTACCATCCATACC	
	TRH P	HEX-TTCCTTCTCCAGGTTCGGATGAGCTACT-BHQ1	

To evaluate the effectiveness of the assay on the detection of actual diarrhea samples, based on the detection results in other studies, we retrospectively selected 164 fecal samples from outpatients with diarrhea from 2016 to 2018 in Wuxi, Jiangsu Province. One to two grams of fecal samples were added to 5 ml of liquid Carry-Blair transport medium and mixed. The samples were delivered to the laboratory at room temperature and frozen to -80°C within 24 hours.

### Primers and Probes

The primers/probes designed for *V. cholerae* (the reaction BDM-VC) targets the genes *ompW*, *ctxA*, *rfbN* (specific for the O1 serogroup), and *wbfR* (specific for the O139 serogroup). The reaction for *V. parahaemolyticus* (BDM-VP) targets the gene *toxR* and the toxin coding genes *tdh* and *trh.* In addition, each reaction contained a pair of primers and a probe targeting *yaiO* gene of *E. coli* as a sample process control (SPC).

All the targeted gene sequences were based on alignments of available sequences deposited in nr database of NCBI (https://www.ncbi.nlm.nih.gov/nucleotide/). All primers and probes were designed using Beacon Designer V8.20, and were synthesized by Sangon Biotech (Shanghai, China). The NCBI BLASTn was used to check the in silico specificity and sensitivity.

### Testing Procedures on the BD MAX Open System Platform

During sample processing, 50 μl of each sample was added into the sample buffer tubes (SBTs) of the BD MAX ExK TNA-2 extraction kit (IDS, BD). SBTs were covered with a cap, vortexed, and placed into the sample rack. Extraction reagent strips of the ExK TNA-2 kit were supplemented with the 2 × PCR master mix of BDM-VC in position 2, 2 × PCR master mix of BDM-VP in position 4, and 25 μl of deionized water in position 3. The positions 2, 3, and 4 are specified in the product manual of ExK TNA-2 kit. The 2 × PCR master mix contained 600 nM of each primer, 200 nM of each probe, 5 μl of 5 × HR qPCR Master Mix (Huirui, Shanghai, China), and deionized water to complete a 12.5 μl final volume. The extraction strip was placed into the testing rack and then the test started running. Nucleic acid from 600 μl of liquid in the SBT was extracted and added to the conical tube containing 25 μl of deionized water at position 3; 12.5 μl of the mixture at position 3 were added into the conical tubes at positions 2 and 4, respectively. Cycling conditions were as follows: 95°C for 5 min and 40 cycles of 95°C for 15 s, 60°C for 43 s.

For SPC and all targets, the result was considered positive when the cycle threshold (Ct) value was ≤ 35. As long as SPC or any target gene was positive, the test was considered valid; if SPC and all target genes were negative, the test was considered invalid, and repeated testing was required.

### Analytical Test

The analytical sensitivity was evaluated using the positive reference strains *V. cholerae* N16961, ATCC 51394, and *V. parahaemolyticus* VP8. Strains were cultured on LB agar and incubated overnight at 37°C. The bacterial lawn was picked into a centrifuge tube with a pipette tip, washed twice with 0.9% sodium chloride solution (normal saline), and then made into a 2.5 McFarland (BD PhoenixSpec, NJ, USA) suspension with normal saline. This was followed by the preparation of six 10-fold dilutions. Ten microliters of each dilution was mixed into 40 μl of healthy human stool samples, which were then transferred to the SBT of the ExK TNA-2 kit for detection on the BD MAX platform. The bacterial concentration of each dilution was calculated by colony count on LB agar. When a standard curve was plotted, Ct values obtained from each dilution were graphed on the y-axis versus the log of the bacterial concentration in artificially spiked stools on the x-axis. The amplification efficiency (E) was calculated from the slope of the standard curve according to the equation: E = 10^-1/slope^-1.

When determining the limit of detection (LoD) of the established assay for each target gene, the 10^-4^ dilution of the bacterial suspension was serially diluted twice in normal saline. As above, 50 μl of artificially spiked stools (containing 10 μl of diluted bacterial solution and 40 μl of healthy human stool) was added to the SBTs and tested on the BD MAX platform. Each dilution was repeated 10 times. When all 10 replicates could be detected, the lowest bacterial concentration in the spiked stool was recorded as the LoD value of the target.

### Analysis of Clinical Samples

Cryopreserved fecal samples, which were collected from diarrheal patients from 2016 to 2018 in Wuxi, Jiangsu Province, were melted at room temperature, and 50 μl of each sample was added into SBT for detection with both BDM-VC and BDM-VP on the BD MAX platform. When the internal control SPC and all target genes were negative, the test was invalid and needed to be repeated. In the repeated detection of invalid samples, three tests were performed in parallel to detect samples, *E. coli* ATCC25922 which could be used as the SPC template, and the mixture of samples and *E. coli* ATCC25922; if the Ct value of SPC increased with the addition of sample in SBT, it was speculated that there is an amplification inhibitor in the nucleic acid of the sample.

Clinical samples were detected in parallel with conventional singleplex real-time PCR assays. The template nucleic acids were extracted from 200 μl of fecal samples using a bacterial genomic DNA extraction kit on the automatic purification system NP968 (Tianlong, Xi’an, China). Species-specific genes of *V. cholerae* and *V. parahaemolyticus* were detected using commercial real-time PCR kits (X-ABT, Beijing, China) in accordance with the manufacturer’s instructions. The virulence genes (*ctxA*, *tdh*, and *trh*) were screened with the primers and probes in **Table 2** in a 20 μl of reaction mixture containing 1 μl of DNA template, 200 nM primers, and 200 nM probes under the following conditions: 95°C for 30 s; 40 cycles of 95°C for 5 s, and 60°C for 40 s. The tests were carried out with a CFX96 system (Bio-Rad, Hercules, CA). Results were considered positive when Ct ≤ 35.

### Statistical Analysis

The overall percent agreement (OPA), positive percent agreement (PPA), and negative percent agreement (NPA) were calculated (Institute, 2008) to evaluate the consistency between the results of BDM-VC/VP and the real-time PCR assays. Cohen’s unweighted *kappa* (Kundel and Polansky, 2003) and the 95% confidence intervals (95% CIs) of the *kappa* value were calculated with SPSS version 23.0 (IBM Corp., Armonk, NY, USA).

## Results

### Analytical Sensitivity and Specificity

**Figure 1:**
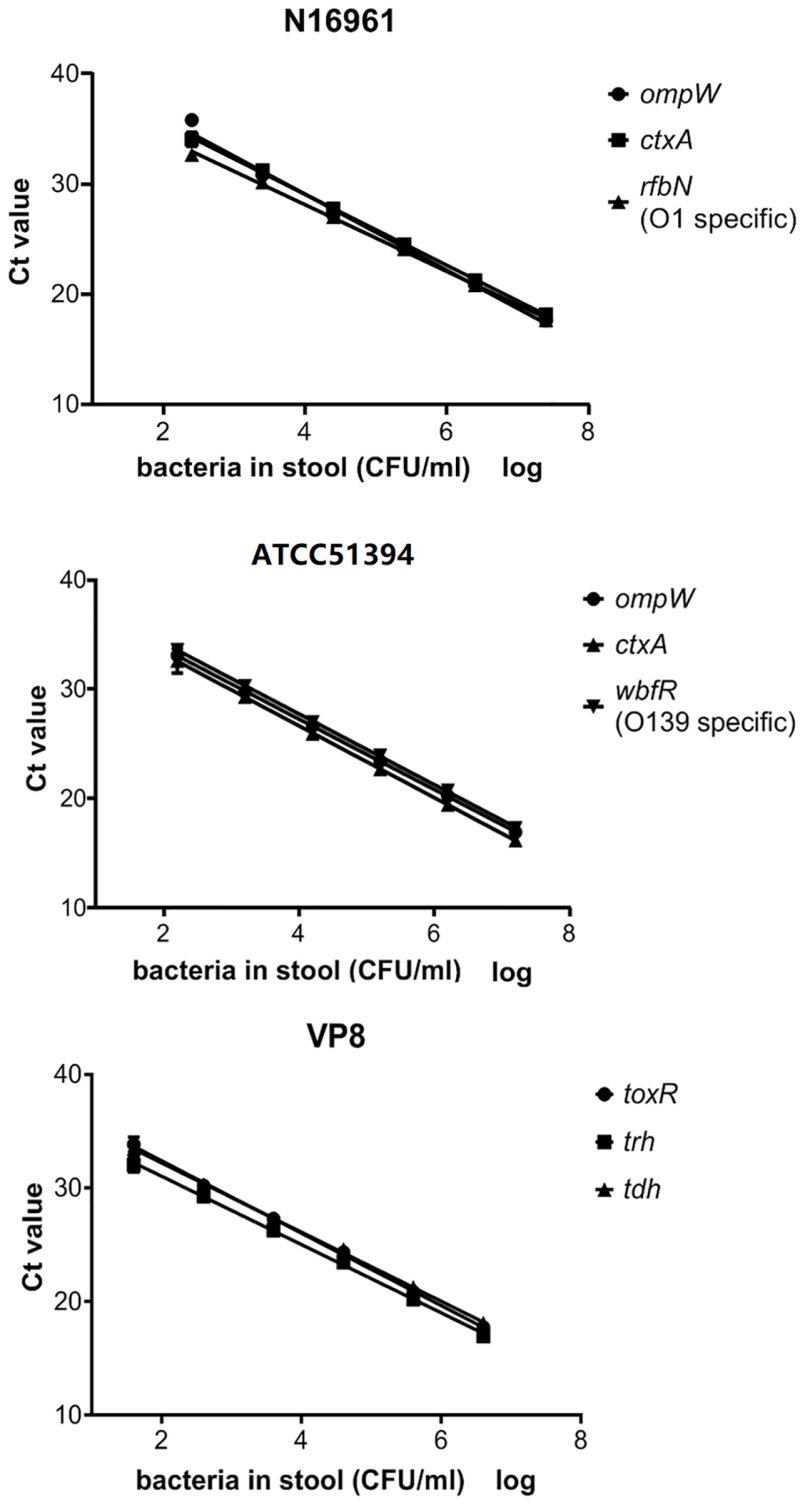
The standard curve of the BD MAX assay for the detection of each gene in artificial samples. Ct values obtained from each dilution were graphed on the y-axis versus the log of the bacterial concentration in artificially spiked stools on the x-axis.

The amplification efficiency and sensitivity of the BD MAX assay for each target gene were evaluated with healthy human stools spiked with serial dilutions of positive reference strains. When the concentration of the strains in the spiked stool samples was 10^2^–10^7^ CFU/ml for *V. cholerae* (N16961 or ATCC51394) and 10^1^–10^6^ CFU/ml for *V. parahaemolyticus*, the standard curves showed good linearity (**Figure 1**), the coefficients of determination R^2^ were all above 0.99, and the amplification efficiency of each target gene was 95.0–115.3% (**Table 3**). Spiked stools with higher and lower bacterial concentrations were not analyzed.

**Table 3:** The amplification efficiency and sensitivity of the BD Max assay for each target gene.

Reaction	Strains spiked in stool	Targeted genes	R^2^	Amplification efficiency	LoD (CFU/ml stool)
BDM-VC	N16961	*ompW*	0.993	95.0%	327.5
		*ctxA*	0.998	104.2%	655
		*rfbN*	0.998	113.8%	655
BDM-VC	ATCC51394	*ompW*	0.999	104.6%	195
		*ctxA*	0.996	101.8%	195
		*wbfR*	1.000	103.7%	780
BDM-VP	VP8	*toxR*	0.998	106.1%	46
		*trh*	0.998	115.3%	46
		*tdh*	0.999	112.8%	184

R^2^, coefficient of determination; LoD, Limit of Detection.

The LoD of BDM-VC for target genes of *V. cholerae* was 328–655 CFU/ml and 195–780 CFU/ml when analyzed with stools spiked with N16961 (O1 serogroup) and ATCC51394 (O139 serogroup), respectively. The BDM-VP reaction was more sensitive, and the LoD of the three target genes was 46–184 CFU/ml.

Specificity of the BD MAX assay was confirmed by testing a panel of strains (**Table 1**). All 22 V*. cholerae* isolates were *ompW* positive by BDM-VC; 11 of these were positive for *rfbN*, 11 were positive for *wbfR*, and 18 were *ctxA* positive. In the detection of 19 V*. parahaemolyticus* and 22 non-target strains, no amplification of BDM-VC was observed except for *ctxA* positive in *V. mimicus* SX-4. All 19 V*. parahaemolyticus* isolates were *toxR* positive by BDM-VP; 10 of these were positive for *tdh*, and one was positive for both *tdh* and *trh*. In the detection of 22 V*. cholerae* and 22 non-target strains, no amplification of BDM-VP was observed. The detection results of BDM-VC/VP were consistent with the singleplex real-time PCR assays (**Table 1**) and showed high specificity.

### Clinical Validation of the BD MAX Assay

In order to evaluate the detection efficiency of the established method for clinical samples, 164 diarrheal fecal samples were selected and detected by the BD MAX assay and conventional real-time PCR respectively, and the results were compared (**Table 4**). BDM-VC detected 7 samples positive for non-O1/non-O139 and non-toxigenic *V. cholerae*. The real-time PCR assays detected 7 samples positive for non-O1/non-O139 and non-toxigenic *V. cholerae*. However, there were two samples with inconsistent results; one was positive by real-time PCR but negative by BDM-VC, and the other was just the opposite, resulting in a *kappa* value of 0.85 (95% CIs, 0.65–1.06) for *V. cholerae.* The *ompW* gene of the former (negative by BDM-VC) was sequenced, and a point mutation was found at the binding site of the BDM-VC probe. The latter was *ompW* positive with a Ct value of 34.4 by BDM-VC. In the repeated real-time PCR tests for *V. cholerae*, the Ct values were 36.1 and 37.1, which were considered negative because they were higher than the threshold of Ct 35. Considering that a strain of *V. cholerae* was isolated from this sample, the false negative of real-time PCR might be mainly due to the low concentration of the strain in the feces.

**Table 4:** Consistency of the BD Max assay and conventional real time PCR results.

		Conventional real-time PCR assay		Consistency between the results			
		+	-	OPA	PPA	NPA	*kappa* (95% CIs)
**BDM-VC**							
*ompW*	+	6	1	98.8%	85.7%	99.4%	0.85 (0.65-1.06)
	–	1	156				
*ctxA, rfbN* and wbfR	+	0	0	100.0%	NA	100.0%	NA
	–	0	164				
**BDM-VP**							
*toxR* & *tdh*	+	29	0	100.0%	100.0%	100.0%	1.00 (1.00-1.00)
	–	0	135				
*trh*	+	2	0	99.4%	66.7%	100.0%	0.80 (0.40-1.19)
	–	1	161				

OPA, overall percent agreement; PPA, positive percent agreement; NPA, negative percent agreement; 95% CIs, 95% confidence intervals; NA, not available.

BDM-VP detected 29 V*. parahaemolyticus* positive samples, 27 positive for *toxR/tdh* and two positive for *toxR/tdh/trh*. The same 29 positive samples were detected by real-time PCR assays, but the results of *trh* in one sample were different: BDM-VP was negative and real-time PCR was positive. By sequencing the amplified products of real-time PCR, it was confirmed that the sample was *trh* positive. Because the target fragments of BDM-VP and real-time PCR on *trh* gene did not overlap each other and the sequence of BDM-VP target fragment was not obtained, it could not be determined whether the false negative was caused by mutations in primers or probe binding sites. The *kappa* values of BDM-VP and the real-time PCR assays were 1.00 (95% CIs, 1.00–1.00) for *V. parahaemolyticus* and *tdh*, and 0.98 (95% CIs, 0.94–1.02) for *trh* (**Table 4**).

Eight of the 164 samples were negative for all targets including SPC by BDM-VC and BDM-VP, and the results were the same in repeated tests. They were also negative by conventional real-time PCR. The template of SPC (that is, the nucleic acid of *E. coli*) was directly added to the reactions of BDM-VC and BDM-VP. When the nucleic acid of the sample was not included in the reaction (control), the Ct value of SPC was 26.4–26.8. When the nucleic acid of the sample was contained in the reaction, the Ct value of SPC was (1) 25.9–27.4 in six samples, close to the control; (2) significantly increased in one sample, 36.2 in BDM-VC and 35.9 in BDM-VP; and (3) still negative in one sample. It was suggested that there were amplification inhibition factors in the nucleic acids extracted from the last two samples.

## Discussion

*V. cholerae* and *V. parahaemolyticus* are important intestinal pathogens of public health concern. In this study, an automated multiplex assay was established on a BD MAX platform to detect *V. cholerae* and *V. parahaemolyticus* as well as their important virulence genes and serogroup-related genes directly from clinical human fecal specimens. We evaluated the sensitivity and specificity of this assay and evaluated its application in clinical diarrhea samples.

In the analytical performance evaluation, the developed assay showed high sensitivity and specificity. The analytical specificity of the assay was established using a group of *Vibrio* isolates from different species as well as other organisms. No cross-reactivity, false positives, or false negatives were observed, even when species closely related to *V. cholerae* and *V. parahaemolyticus* were tested. The assay was shown to be very sensitive with a LoD as low as 46–780 CFU/ml and to have high PCR efficiency between 95.0 and 115.3% with spiked fecal samples. The sensitivity of this assay was close to the real-time PCR methods used to screen other pathogens in fecal samples (Raphael and Andreadis, 2007; Lin et al., 2008; Luna et al., 2011; Kouhsari et al., 2019).

In the detection of 164 retrospective stool samples, the developed assay on BD MAX achieved results comparable to the conventional real-time PCR assays. The inconsistent results of the two samples in the detection of *V. cholerae* showed that the BD MAX assay was more sensitive, but also revealed the defect that the probe sequence does not match the target sequence occasionally. BD MAX was also found to have an occasional false negative in the detection of the *trh* gene, which was speculated to be related to the diversity of *trh* sequences (Kishishita et al., 1992). In addition to the detection of *V. cholerae* and *V. parahaemolyticus* in clinical samples, the BD MAX assay screened important virulence genes and serogroup-specific genes, providing more information in one test regarding pathogens of interest than the comparison conventional assays.

The BD MAX assay contains an internal control SPC (a pair of primers and a probe designed according to the sequence of *E. coli* gene *yaiO*), which can monitor the processes of fecal nucleic acid extraction and PCR amplification. The failure of the SPC detection (unresolved results) could be caused by inhibitory substances in the stool samples or reagent, or potential instrument failure. Evaluation of the commercially available BD MAX EBP assay revealed the rate of unresolved results was 2.4–8.03% (Harrington et al., 2015; Knabl et al., 2016; Simner et al., 2017). In this study, eight samples (4.9%) were unresolved. This proportion is consistent with literature reports. The negative of SPC in two samples (1.2% of the total and 25% of the unsolved) was due to the existence of amplification inhibition factors, while in other samples it might be due to the low concentration of *E. coli*, which might be caused by insufficient preservation of fecal samples when collected or during transportation, or by the degradation of nucleic acid during long-term preservation. When the developed assay was used for sample detection in this study, no exogenous SPC template was added to the samples or reaction system, which could monitor not only the amplification inhibitory factors in the sample but also the quality of the sample.

This assay has several limitations. The assay is incapable of detecting pathogens with mutations in the sequence of the binding sites of primers and probes. Like other PCR-based methods, the assay can amplify the DNA of dead bacteria in the sample, so a positive result does not necessarily indicate an active infection.

This multiplex assay was carried out on a BD MAX open system, a fully integrated sample-to-answer platform that performs both nucleic acid extraction and real-time PCR. Twenty-four samples can be detected at the same time. It took no more than 15 min hands-on time and less than 3 h for results, compared to conventional culture methods that would take days. Minimum manual operation can reduce potential human error, contamination, and potential biohazard to laboratory workers. This developed assay demonstrates potential promise to be useful in clinical settings routinely for detecting two of the most clinically important *V. cholerae* and *V. parahaemolyticus* species in public health.

## Data Availability Statement

The original contributions presented in the study are included in the article/supplementary material. Further inquiries can be directed to the corresponding author.

## Ethics Statement

The studies involving human participants were reviewed and approved by Ethics Committee of National Institute for Communicable Disease Control and Prevention, China CDC. Written informed consent to participate in this study was provided by the participants’ legal guardian/next of kin.

## Author Contributions

ZL: paper writing and data analysis. HXG: the test of clinical diarrheal samples. WW: evaluation of the analytical sensitivity and specificy of the multiplex assay. HG and WF: the collection of clinical diarrheal samples. JL, BD, and HZ: the identification of strains used in the study. BK: study design. JZ: study design, data analysis, and paper review. All authors contributed to the article and approved the submitted version.

## Funding

This study was supported by National Science and Technology Major Project of China from the Ministry of Science and Technology of the People’s Republic of China (2018ZX10712001-014 and 2018ZX10305409-003-003) and Wuxi Health Commission (T202017). The BD MAX instrument was provided by BD China free of charge, and no financial support was received from BD or any other commercial entities.

## Conflict of Interest

A PCT patent application has been filed in China.

The authors declare that the research was conducted in the absence of any commercial or financial relationships that could be construed as a potential conflict of interest.
